# Mediators and moderators in voluntary turnover intention: a scoping review in the public service

**DOI:** 10.3389/fpsyg.2025.1631551

**Published:** 2025-10-28

**Authors:** Ana Cláudia A. M. Silva, Elaine R. Neiva, Daniel Pereira Damasceno, Felipe Orsini F. B. Ferreira

**Affiliations:** Department of Social, Labor, and Organizational Psychology, Institute of Psychology, University of Brasília, Brasília, Brazil

**Keywords:** voluntary turnover intention, moderator, mediation, scoping review, HRM

## Abstract

**Background:**

Recent meta-analyses present the main predictors of voluntary turnover intention (VTI) and propose the investigation of mediators and moderators as a way to enhance the explanation of the relationship.

**Objectives:**

This study conducts a scoping review of the main moderating and mediating variables in studies on VTI, focusing on the public servants in the executive branch. The review aimed to fill gaps by including more studies from developing and Asian countries, identifying the main mediating and moderating variables that can help explain VTI from the initial thought to the actual departure. Method: The PRISMA protocol for scoping reviews was followed. Online database searches were conducted to identify empirical articles published in the last 20 years, from which 51 publications were selected.

**Results:**

Forty-five different variables were identified, grouped into six different dimensions: (a) motivation, attitudes, and behaviors related to work; (b) person-organizational context relationship; (c) organizational characteristics and context; (d) personal conditions related to work; (e) human resource management practices; and (f) demographics. Moderating variables were found in 15 studies, mediating variables in 26 studies, and 10 studies evaluated both relationships.

**Conclusion:**

Seven variables showed a complete mediating effect, which can strongly contribute to explaining the path leading to VTI. These variables are person-organization fit, goal clarity, intrinsic motivation, organizational prestige, leader-member exchange, job satisfaction, and organizational support. Additionally, important moderators related to human resource management practices were detected. Human Resources (HR) departments within public sector organizations can leverage the key mediating and moderating variables from this review to periodically assess employee turnover and VTI.

## 1 Introduction

Both public and private organizations are concerned with turnover or the loss of an employee, especially when the departing individual is a key person. The departure of this individual can lead to a loss of institutional memory (Verma and Kesari, 2020), the need for personnel replacement, higher costs associated with hiring and training new professionals (Moynihan and Pandey, 2007; Sharma and Stol, 2020), absenteeism, presenteeism, or demotivation (Chang et al., 2013), increased workload for remaining employees, and work disruption (Diógenes et al., 2016).

Every organization experiences a certain level of turnover, represented by the ratio of members who leave the organization during a specific period, typically expressed as a percentage (March and Simon, 1965). Turnover becomes problematic when the percentage of individuals leaving is high (Kim and Fernandez, 2017). Organizational turnover is classified as either involuntary or voluntary. Involuntary turnover is characterized by departures initiated by the organization (Heavey et al., 2013), such as dismissal or, in the case of an employee’s death. Conversely, voluntary turnover refers to departures initiated by employees themselves, stemming from their own interest and at their request—that is, it possesses a volitional characteristic (Mobley, 1982; Hom and Griffeth, 1995).

Much of the research in this field focuses on the voluntary aspect of turnover, as it is often considered more detrimental to the organization and occurs more frequently (Hom and Griffeth, 1995; Lambert and Hogan, 2009; Kim and Fernandez, 2017), and, more specifically, on voluntary turnover intention (VTI). The intention to engage in a behavior is the best predictor of actually performing that behavior (Ajzen and Fishbein, 2000). VTI is an immediate precursor to the behavior of leaving, i.e., the desire to leave the organization, an antecedent to actual departure (Mobley, 1977; Mobley et al., 1978). VTI is distinct from other similar constructs, such as negative behaviors detrimental to others or the organization, disengagement behaviors, and work avoidance behaviors, according to a recent meta-analysis (Carpenter and Berry, 2017).

Although high VTI does not always equate to actual employee departure, cognitions related to thoughts of leaving demonstrated the strongest correlations with actual turnover in a recent meta-analysis (Rubenstein et al., 2018). VTI is the final stage of a multi-step decision-making process that can culminate in an employee’s departure (Campbell and Im, 2016). Analyzing VTI can offer several benefits to organizations, such as: (a) identifying the percentage of employees who exhibit VTI; (b) identifying the key variables or factors that influence VTI; (c) enhancing human resource management practices to address the key identified factors and variables influencing VTI; and (d) reducing organizational costs associated with actual turnover (Morrell et al., 2004; Moynihan and Landuyt, 2008; Jung, 2014; Hom et al., 2017).

Theories explaining employee departure originated in the late 20th century, primarily inspired by motivational causes, particularly job dissatisfaction (Mobley, 1977; Mobley et al., 1978; March and Simon, 1979). These initial theories were subsequently expanded over more than a century of research (Hom et al., 2017), elucidating the understanding of distal and proximal antecedents to the phenomenon of voluntary employee departure from their work environment (Porter et al., 1976; Steers and Mowday, 1981; Mobley, 1982; Hom and Griffeth, 1991). Subsequent theoretical developments indicate that departure involves a process with various potential pathways, following an initial trigger by thoughts of leaving (Mitchell and Lee, 2001; Hom et al., 2017).

Lee and Mitchell’s (1994) unfolding model of turnover, one of the most cited in the literature, posits an interconnection among cognitions, affect, and thoughts. This process is driven by various interrelated variables that, by influencing one another, lead an individual to the decision to stay with or leave an organization. Consequently, the path from the initial thought of leaving to actual turnover involves antecedent and predictor variables, as well as mediating and moderating variables (Lee and Mitchell, 1994; Mitchell and Lee, 2001; Allen and Hancock, 2024). Despite representing a significant advancement, none of these models provides clarity on what the main variables are. In this regard, two meta-analyses (Rubenstein et al., 2018; Hur and Abner, 2024) identify the primary antecedent and predictor variables. However, both meta-analyses do not specify which variables act as mediators or moderators in the relationships.

Antecedent and predictor variables with the largest effect sizes that can lead to voluntary departure fall into the following main categories: (a) work-related motivation, attitudes, and behaviors; (b) person-context fit; (c) organizational characteristics and context; (d) work-related personal conditions; (e) human resource management practices; (f) demographics; (g) external labor market factors; and (h) disengagement attitudes (Rubenstein et al., 2018; Hur and Abner, 2024).

The meta-analysis by Rubenstein et al. (2018) primarily comprises studies conducted in private companies. The meta-analysis by Hur and Abner (2024) focuses on the public sector. A key limitation identified in both meta-analyses is their reliance on research primarily from the United States of America (USA) and Western European countries, with limited inclusion of other countries and cultures. These findings may not be generalizable to developing countries, such as Brazil, and nations in Africa and Asia. There is a prevalence of a Western perspective, likely reflecting a predominantly White viewpoint and originating from more developed nations.

Hur and Abner (2024) also highlight a research gap concerning the exploration of moderators to explain additional variance in the relationship between predictors and VTI. An analysis evaluating moderating variables examines whether the relationship between two variables is influenced by a third variable (Hayes, 2018). Moderation indicates when or for whom certain effects are stronger or weaker. Research is already advancing in this direction by focusing on complex relationships; for instance, a study with 250 employees from public and private organizations in Belgium found that ethical leadership moderated the relationship between frequent changes and VTI, weakening this association (Babalola et al., 2016). A high psychological climate was found to moderate the relationship between low affective commitment and VTI in a study in Ghana (Gyensare et al., 2017).

Hur and Abner (2024) also suggest that future research should simultaneously examine the strongest attitudinal predictors of turnover to determine reciprocal relationships and uncover mediating influences. Mediating variables intervene in the process or are mechanisms through which an independent variable influences a dependent variable (Hayes, 2018). A mediator is positioned between a key antecedent variable and VTI, meaning there is an indirect effect.

Many studies explore the mediation of attitudes due to their capacity to shape affect, behaviors, and cognitions regarding the work environment (Grama and Todericiu, 2016). A study with graduates of a hospitality business program and technology industry professionals, respectively, found that career commitment partially mediated the relationship between challenging work and VTI, and fully mediated the relationship between social support and VTI (Walsh, 2016; Lin, 2020). A study involving 189 nurses in Portugal found that a sense of calling fully mediated the relationship between challenging job demands and VTI (Esteves and Lopes, 2017).

Understanding both moderators and mediators influencing VTI is essential for developing more targeted and effective organizational strategies tailored to different contexts and employee subgroups. This contributes to an in-depth understanding of the turnover process, aids in reducing turnover, and supports talent retention. Identifying the primary reasons why individuals consider leaving their current workplace, and subsequently seeking to mitigate these factors, is far more effective than dealing with the problems and financial costs associated with actual employee departures (Chung and Jeon, 2020).

Thus, the primary objective of this literature review is to identify the key moderating and mediating variables that appear in studies where VTI is assessed in public organizations within the executive branch. By restricting the scope to the executive branch, the aim is to subsequently replicate these key variables in predictive models of VTI within the same branch of government in Brazil.

This review will also seek to broaden the scope to include studies from developing countries, drawing parallels with the variables highlighted in the meta-analyses (Rubenstein et al., 2018; Hur and Abner, 2024) and identifying those variables that have been studied according to the local context of each study. It will be important to detect instances where the same variable can function as either a mediator or a moderator, as this depends on “how the phenomenon under investigation is conceptualized and subsequently tested” (Hayes, 2018, p. 9).

## 2 Methods

A scoping review methodology was adopted (Arksey and O’Malley, 2005; Pham et al., 2014) to identify moderating and mediating variables in research on VTI, addressing a gap identified in the study by Hur and Abner (2024). This review followed five main stages: (1) identifying the research question, (2) identifying relevant studies, (3) study selection, (4) charting the data, and (5) collating, summarizing, and reporting the results (Arksey and O’Malley, 2005; Levac et al., 2010).

### 2.1 Research question

The research question guiding this study was: “What are the key mediating and moderating variables, along with their respective instruments, found in studies on VTI among public servants working in the executive branch?” This question addresses the specific population, construct, context, and research design (Tricco et al., 2018).

### 2.2 Identification of relevant studies

On 18 February 2024, searches were conducted in the electronic databases EBSCO, Web of Science, American Psychological Association (APA), Latin American and Caribbean Health Sciences Literature (LILACS), and PubMed. These databases index research in the fields of administration and business, health, psychology, and education—the main knowledge areas that study VTI. The searches were limited to the inclusion of the English word “turnover” in the title and the English word “public” in the abstract of the articles. For all fields, the exclusion of the English word “private” was requested. The search strategy was: [(TI = (“turnover”)) AND AB = (“public”)] NOT ALL = (“private”). Filters were applied to restrict results to peer-reviewed scientific articles published within the last 20 years.

The following inclusion criteria were adopted: (1) quantitative articles; (2) VTI had to be the predicted or dependent variable; (3) the sample had to consist of public servants within the executive branch in their respective countries. Articles that did not meet these criteria were excluded. The identification of mediating and moderating variables was deferred to the full-text reading stage, as it was found that in older articles, this information was often absent from the abstracts.

#### 2.2.1 Study selection

The identification and selection process for relevant articles was conducted by two independent reviewers. In cases of disagreement between them, a third reviewer, an expert in the field, made the final decision. The two independent reviewers initially screened the titles and abstracts of all articles to select those for the eligibility assessment. Articles that advanced to the eligibility assessment were read in full, and those lacking mediating or moderating variables were excluded at this stage. Also excluded were articles where the sample did not consist of public servants from the executive branch, those that did not feature VTI as a dependent or predicted variable, articles that could not be retrieved, and articles in Chinese, Japanese, or Serbian, which could not be read. The Kappa coefficient calculation (Landis and Koch, 1977) indicated almost perfect agreement between the reviewers, both for article inclusion in the screening phase (κ = 0.94, *p* < 0.001) and for inclusion in the eligibility phase (κ = 0.89, *p* < 0.001).

The risk of bias in each article selected for eligibility was assessed using the Risk of Bias Utilized for Surveys Tool (ROBUST), which evaluates presence or absence (yes or no). Of the eight specified types of risk of bias, seven were used. The eight types of biases considered refer to: (1) sample representativeness; (2) participant recruitment method; (3) percentage of excluded participants; (4) final sample size; (5) presentation of sociodemographic variables; (6) reliability of the measures used; and (8) data administration (Nudelman and Otto, 2020). Criterion (7), controlled data collection environment, was not used because the research objective is to analyze organizational environments, meaning there is no control of conditions as in a laboratory setting.

**FIGURE 1:**
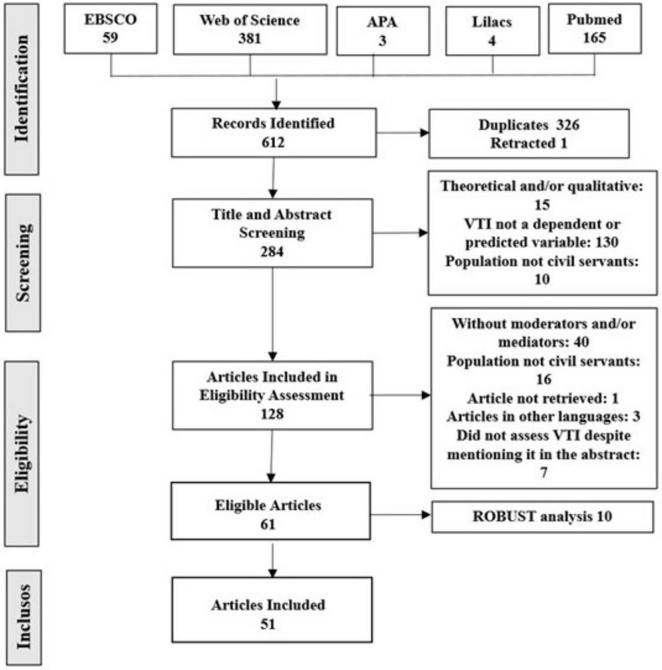
Stages of the literature review.

The ROBUST was completed by three independent reviewers, and in cases of disagreement among them, a fourth reviewer was consulted to resolve discrepancies. Compliance with the criteria was coded as 1 for “yes” and 2 for “no.” The Kappa calculation indicated agreement ranging from substantial to almost perfect (0.73 ≤ κ ≤ 0.98, *p* < 0.001; 95% CI). Supplementary Appendix C shows the bias analysis performed, and all articles that did not meet a minimum of four criteria were excluded. Figure 1 shows each of the stages, the number of articles, the exclusion criteria, and the number of articles eliminated. All files for this review are available at https://osf.io/eb3uf/files/osfstorage.

### 2.3 Data extraction and organization

The reviewers who participated in the article selection stage also worked independently on extracting relevant data from the articles. In cases of disagreement between the reviewers, a third reviewer was consulted (the same one from the previous stage). The following information was extracted from the articles: (1) mediating and moderating variables present in the studies; (2) instruments used (type, measurement method, and sample item); (3) country of the authors and of the study; (4) study period; and (5) sample (size, type, and field of work).

## 3 Results

**FIGURE 2:**
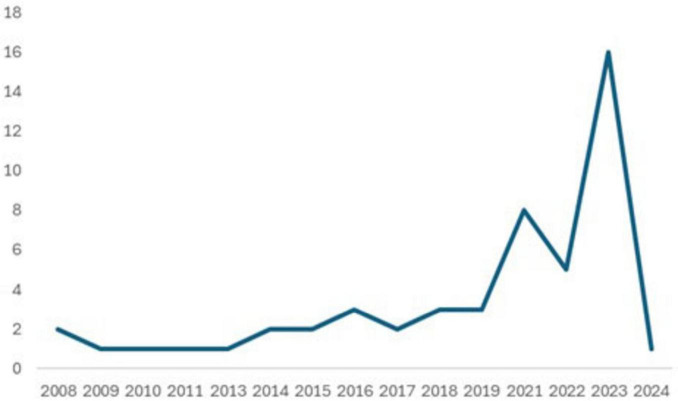
Number of articles published per year.

This section presents the compiled, summarized, and reported results. Supplementary Appendix A provides the table with the information extracted from the 51 articles included in this scoping review. The list of excluded articles is provided in Supplementary Appendix B. The risk of bias assessment is provided in Supplementary Appendix C. All appendices are in the Supplementary materials. There was an increase in the number of studies from 2016 onward, with a peak in 2023 with the publication of 19 articles, as shown in Figure 2. The topic of VTI has been gaining prominence in the public service. The year 2020 coincided with the COVID-19 pandemic, which explains the drastic reduction; data for 2024 reflects only the first 2 months of the year.

**FIGURE 3:**
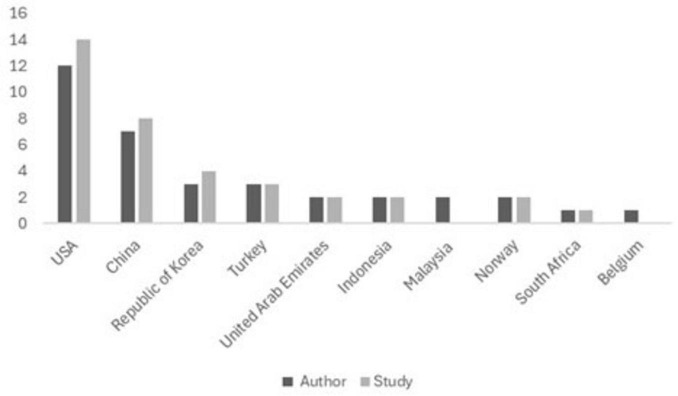
Leading countries by author origin and study location.

Only 11 articles referenced by Hur and Abner (2024) are included in this scoping review (Pitts et al., 2011; Campbell et al., 2014; Ertürk, 2014; Kim, 2015; Caillier, 2016a,b; Campbell and Im, 2016; Kim and Fernandez, 2017; Shim et al., 2017; Jin et al., 2018; Sabharwal et al., 2019). The adopted search strategy enhanced the inclusion of Eastern and developing countries. Nevertheless, a prevalence of the USA is still evident, accounting for the most authors and investigated samples, as can be observed in Figure 3, which provides a breakdown of the leading countries by author origin and study location.

In addition to the countries shown in Figure 3, the following countries are also represented with authors and samples: Canada, Egypt, Ethiopia, Ghana, India, Nigeria, Pakistan, Thailand, and Vietnam. The following countries appear only with authors conducting research in other nations: Saudi Arabia, Bangladesh, Cyprus, Spain, Portugal, the United Kingdom, and Russia. The following countries have groups of public servants investigated in studies but do not have authorship in the analyzed studies: Australia, Bhutan, Iraq, Jordan, and Kuwait.

Only two studies featured a longitudinal design (Mullins et al., 2021; Chordiya, 2022a). Convenience sampling accounted for 49% of the articles, and random sampling for 51%. Regarding the public servants’ field of work, the studies were distributed as follows: multiple areas (37.25%), education (17.65%), health (13.73%), others (13.73%), government agencies (9.80%), inspection/regulation (3.92%), public safety (1.96%), and not described (1.96%).

Fifteen studies included only moderating variables, 26 studies assessed mediating relationships, and 10 assessed both. In total, there are 45 different variables, which were grouped based on the divisions proposed Hur and Abner (2024), Rubenstein et al. (2018) meta-analyses: (a) work-related motivation, attitudes, and behaviors; (b) person-organizational context fit; (c) organizational characteristics and context; (d) work-related personal conditions; (e) human resource management practices; and (f) demographics (Hur and Abner, 2024; Rubenstein et al., 2018). These authors compile the categories from the literature (Steers and Mowday, 1981; Lee and Mowday, 1987; Holtom et al., 2008). The mediating and moderating variables extracted from the articles were then classified by each judge according to the previously defined categories. Table 1 presents the grouping with the largest number of variables (12) aimed at measuring individuals’ motivation, attitudes, and behaviors toward their work.

**TABLE 1:** Work-related motivation, attitudes, and behaviors.

Variables	Study (country)	Field of work	Med	Mod	Category %
1. Job satisfaction	South Africa	Agencies	–	1	44.00%
	China	Education	2	–	–
		Multiple areas	2	–	–
	United Arab Emirates	Multiple areas	1	–	–
	USA	Agencies	1	–	–
		Education	1	–	–
		Multiple areas	1	–	–
	Kuwait	Education	1	–	–
	Thailand	Multiple areas	1	–	–
2. Public service motivation	China	Multiple areas	–	1	16.00%
		Not described	–	1	–
	Republic of Korea	Multiple areas	–	1	–
		Others	–	1	–
3. Negative affect	China	Multiple areas	1		4.00%
4. Self-efficacy	USA	Multiple areas	1		4.00%
5. Change-oriented organizational citizenship behavior	Republic of Korea	Outros	1	–	4.00%
6. Followership behavior	USA	Education	1	–	4.00%
7. Career commitment	China	Multiple areas	1	–	4.00%
8. Incivility	Indonesia	Multiple areas	–	1	4.00%
9. Employee loyalty	Jordan	Multiple areas	1	–	4.00%
10. Intrinsic motivation	Iraq	Education	1	–	4.00%
11. Life satisfaction	Vietnam	Multiple areas	1	–	4.00%
12. Training satisfaction	Jordan	Multiple areas	1	–	4.00%
Total			19	6	

Med, mediator; Mod, moderator.

Job satisfaction was the most common variable, appearing in 11 studies, and it served as a moderator once in the relationship between talent management practices and VTI (Barkhuizen and Gumede, 2021). Job satisfaction appeared as a full mediator in three studies in the relationship with VTI and the following variables: principal support, organizational learning culture, and person-organization fit (Liu et al., 2010; Lin et al., 2022; Al-Mahdy and Alazmi, 2023).

Job satisfaction appeared as a partial mediator in 6 studies in the relationship with VTI and the following variables: quality of work life, perceived organizational fit, pay equity, empowerment, public service motivation, strategic human capital management, and burnout (Kim and Fernandez, 2017; Jabeen et al., 2018; Jin et al., 2018; Liu et al., 2023; Zhang et al., 2023; Wang et al., 2024; Wesemann, 2024).

Most studies used four items to measure the construct, with five-point Likert-type scales (1 = strongly disagree to 5 = strongly agree). All studies were cross-sectional, and 45.45% used convenience samples (73% of which involved public servants working in multiple fields). Each study either used a different scale or created a specific measure to assess job satisfaction.

The second most studied variable in the first category is public service motivation, assessed as a moderator in Asian countries (China and South Korea). Public service motivation weakens the strength of the relationship between VTI and the following variables: occupational stress (challenge and hindrance stressors), perceived over qualification, emphasis on efficiency, and job demands (Campbell et al., 2014; Shim et al., 2017; Bao and Zhong, 2021, 2023). Scales developed by two authors were featured in an equal number of studies (Perry, 1996, 1997; Wright et al., 2013). The samples had a minimum of 300 participants and a maximum of 979.

The remaining variables appear only once. Intrinsic motivation appeared as a full mediator in the relationship between ethical leadership and VTI in a study in Iraq (Shareef and Atan, 2019). The following variables acted as partial mediators: (a) negative affect (Bao and Zhong, 2023); (b) self-efficacy (Caillier, 2016b); (c) change-oriented organizational citizenship behavior (Campbell and Im, 2016); (d) followership behavior (Jin et al., 2018); (e) career commitment (Li and Xie, 2022); (f) loyalty (Albtoosh et al., 2022); (g) life satisfaction (Nguyen T. D. et al., 2023); and (h) training satisfaction (Albtoosh et al., 2022). Workplace incivility appears as the only moderator in a cross-sectional study in Indonesia (Wirawan et al., 2023).

The second grouping with the largest number of variables represents a dynamic interaction between the individual and the context in which they are embedded within the organization, as presented in Table 2. This category was termed person-organizational context fit (Rubenstein et al., 2018; Hur and Abner, 2024). Person-organization fit was the most researched variable in the second category, with three studies.

**TABLE 2:** Person-organizational context fit.

Variables	Study (country)	Field of work	Med	Mod	Category %
1. Person-organization fit	USA	Multiple areas	–	1	16.67%
		Health	1	–	–
	Pakistan	Education	1	–	–
2. Distributive justice	Republic of Korea	Multiple areas	–	1	16.67%
	USA	Multiple areas	–	1	–
	Indonesia	Inspection/regulation	1	–	–
3. Procedural justice	Republic of Korea	Multiple areas	–	1	11.11%
	USA	Multiple areas	–	1	–
4. Organizational identification	Australia	Multiple areas	–	1	11.11%
	China	Not described	1	–	–
5. Ethical leadership	Vietnam	Multiple areas	–	1	5.56%
6. Leader-member exchange	Turkey	Others	1	–	5.56%
7. Off-the-job embeddedness	United Arab Emirates	Health	–	1	5.56%
8. Organizational trust	Turkey	Others	–	1	5.56%
9. Perceived competence mobilization	Norway	Public safety	1	–	5.56%
10. Servant leadership	China	Multiple areas	–	1	5.56%
11. Trust in supervisor	Turkey	Others	–	1	5.56%
Total			7	11	

Med, mediator; Mod, moderator.

In the first study, person-organization fit appeared as a moderator in the relationship between VTI and abusive supervision (Nguyen N. T. H. et al., 2023). In two studies, person-organization fit acted as a full mediator in relationships between VTI and public service motivation, and work-life balance practices (Bright, 2008; Kakar et al., 2019). All studies were cross-sectional, and most used convenience samples.

The variable distributive justice appeared in three studies: the first as a partial mediator (Supi et al., 2023) and in two others as a moderator (Campbell et al., 2014; Chordiya, 2022a). Procedural justice appeared as a moderating variable on two occasions (Campbell et al., 2014; Chordiya, 2022a).

These justice aspects were assessed simultaneously in a longitudinal study by Chordiya (2022a) with large American samples, using the Federal Employee Viewpoint Survey (FEVS), which in some years recorded over 600,000 responses. Another variable with two studies was organizational identification, which showed partial mediation with Chinese public servants (Bao and Zhong, 2021) and a moderating effect with Australian public servants (Boon et al., 2021).

The remaining variables each feature in only one study. Moderating effects were found for the following variables: (a) organizational trust (Ertürk, 2014); (b) trust in supervisor (Ertürk, 2014); (c) off-the-job embeddedness (Hussain and Deery, 2018); (d) ethical leadership (Nguyen T. D. et al., 2023); and (e) servant leadership (Li and Xie, 2022). Partial mediation was found for perceived competence mobilization (Lai and Kapstad, 2009). Leader-member exchange appears in the same study as a full mediator between information sharing and VTI, and with a partial mediating effect between recognition and VTI (Ertürk, 2014).

The third grouping of variables is related to the work environment, as shown in Table 3, and is termed organizational context (Rubenstein et al., 2018; Hur and Abner, 2024). The variable perceived organizational support (POS) appears in three studies: first, as a full mediator (Ertürk, 2014), and twice as a partial mediator (Liu et al., 2023; Supi et al., 2023). The studies were conducted in Eastern countries, and one author’s scale was the most used (Eisenberger et al., 1986), even with variations in the number of items included (from 6 to 8). All studies were cross-sectional and ranged from 204 to 2,079 respondents.

**TABLE 3:** Organizational context.

Variables	Study (country)	Field of work	Med	Mod	Category %
1. Perceived organizational support (POS)	China	Education	1	–	25.00%
	Indonesia	Inspection/Regulation	1	–	–
	Turkey	Others	1	–	–
2. Perceived supervisor support	Norway	Multiple areas	–	1	16.67%
	Turkey	Health	–	1	–
3. Agency type	USA	Agencies	–	1	8.33%
4. Collaborative culture	USA	Education	1	–	8.33%
5. Goal clarity	USA	Multiple areas	1	–	8.33%
6. Mission valence	USA	Agencies	1	–	8.33%
7. Organizational climate for innovation	Republic of Korea	Multiple areas	–	1	8.33%
8. Turnover system type	USA	Others	1	–	8.33%
9. Perceived organizational prestige	USA	Others	1	–	8.33%
Total			8	4	

Med, mediator; Mod, moderator.

In the third grouping, the second variable with the highest number of studies is supervisor support, which appears as a moderator in cross-sectional studies, in the relationship between VTI and perceived job autonomy and work-family conflict (Dysvik and Kuvaas, 2013; Yucel et al., 2023). The remaining variables in this set appear only once. Organizational climate for innovation appears as a moderator in the relationship between emphasis on efficiency and VTI (Campbell et al., 2014). Also acting as a moderator is agency type in the relationship between being an employee identified as LGBT (lesbian, gay, bisexual, and transgender) and VTI (Sabharwal et al., 2019).

Full mediation occurred with goal clarity (Caillier, 2016b) and perceived organizational prestige (Bright, 2021). The following variables were found as partial mediators: collaborative culture (Sun and Wang, 2017); turnover system type (Strolin-Goltzman et al., 2008); and mission valence (Caillier, 2016a). Commonly, these studies were cross-sectional, with random samples of over 500 American public servants.

Included in the fourth grouping, as shown in Table 4, are variables that address certain aspects of work or the environment with effects on the mental, emotional, and/or physical health of public servants, creating positive or negative impacts. This grouping was termed work-related personal conditions (Rubenstein et al., 2018; Hur and Abner, 2024).

**TABLE 4:** Work-related personal conditions.

Variables	Study (country)	Field of work	Med	Mod	Category %
1. Work engagement	Ethiopia	Education	–	1	38.46%
	Gana	Multiple areas	–	1	–
	Nigéria	Education	1	–	–
	Turkey	Health	2	–	–
2. Burnout	Pakistan	Health	2	–	30.77%
	Republic of Korea	Others	1	–	–
		Inspection/regulation	–	1	–
3. Job insecurity	Indonesia	Multiple areas	–	1	15.38%
4. Occupational stress	Pakistan	Health	1	0	7.69%
5. Psychological distress	USA	Multiple areas	1	–	7.69%
Total			11	2	

Med, mediator; Mod, moderator.

The most researched variable in the fourth grouping was work engagement, with five studies. In the first, it appears as a moderator in the relationship between the psychological impact of the COVID-19 pandemic and VTI (Obuobisa-Darko and Sokro, 2023). Work engagement appeared as a partial mediator in the remaining studies, in the relationship between VTI and the following variables, respectively: POS, transformational leadership, human resource management practices, and work-family conflict (Gadi and Kee, 2020; Bas and Çınar, 2021; Diko and Saxena, 2023; Yucel et al., 2023). Three studies were from African countries, and the most common scale was the 9-item scale by Schaufeli et al. (2006), a five-point Likert-type scale (Schaufeli and Bakker, 2004; Schaufeli et al., 2006). The most frequent sample sizes ranged between 301–400 and 401–500 public servants.

Burnout (exhaustion) was found four times in this grouping, always as a partial mediator, in Eastern countries (Kim, 2015; Shim et al., 2017; Samad et al., 2021; Aman-Ullah et al., 2023). Half of the studies were in the health sector, with samples between 200 and 300 public servants, with a preference for using items from the Maslach Burnout Inventory, employing seven-point Likert-type scales (Maslach and Jackson, 1981; Maslach et al., 2001). Burnout mediated the relationships between VTI and: intrinsic motivation (Kim, 2015), job demands (Shim et al., 2017), and workplace incivility (Samad et al., 2021; Aman-Ullah et al., 2023).

In third place in the fourth grouping is job insecurity, with two studies as a partial mediator in Eastern countries. Both studies had a cross-sectional design and used convenience samples of between 201 and 300 public servants. Job insecurity mediated the relationship between VTI and fear of COVID-19, and citizen incivility, respectively (Kakar et al., 2023; Wirawan et al., 2023). The other variables each have a single study. Psychological distress was a partial mediator of the relationship between VTI and abusive supervision (Nguyen N. T. H. et al., 2023). Occupational stress was a partial mediator of the relationship between VTI and workplace incivility (Samad et al., 2021).

The fifth category includes variables related to how organizations manage their public servants, as shown in Table 5, termed human resource management practices (Rubenstein et al., 2018; Hur and Abner, 2024). Each variable features in a single study and acts as a moderator (Sabharwal et al., 2019; Mullins et al., 2021; Chordiya, 2022a,b; Xu et al., 2023; Wang et al., 2024). Commonly, all studies assessed more than 500 public servants. Three studies used census data from American (FEVS) and Canadian Public Service Employee Survey public servants. One of the studies was longitudinal (Chordiya, 2022a). For all these variables, the moderation hypotheses were corroborated, attenuating VTI.

**TABLE 5:** Human resource management practices.

Variables	Study (country)	Field of work	Mod	Category %
1. Benefits package	China	Health	1	16.67%
2. Career growth opportunities	China	Multiple areas	1	16.67%
3. Flexible work arrangements	Canada	Others	1	16.67%
4. Inclusion quotient	USA	Agencies	1	16.67%
5. Inclusive organizational practices	USA	Multiple areas	1	16.67%
6. Pro-diversity management	USA	Multiple areas	1	16.67%
Total			6	

Med – Mediator; Mod -Moderator.

The smallest grouping was presented in Table 6, termed demographics (Hur and Abner, 2024; Rubenstein et al., 2018). Belonging to the Millennial generation was included here because the cutoff was based on an age limit of 29 years. Three studies were conducted in which the variables acted as moderators (Pitts et al., 2011; Ertas, 2015; Senapaty and Venugopal, 2023).

**TABLE 6:** Demographics.

Variables	Study (country)	Field of work	Mod	Category %
1. Age	USA	Others	1	66.67%
	India	Others	1	
2. Millennial status	USA	Agencies	1	33.33%
Total	3			

Med – Mediator; Mod -Moderator.

**FIGURE 4:**
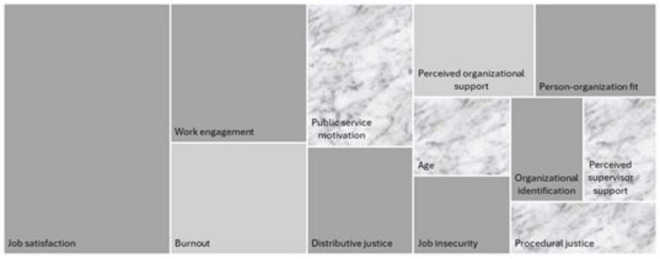
Key variables by type.

A compilation of the main moderating and mediating variables, with their respective scales, measurement methods, and study and sample characteristics, can be found in Supplementary Appendix A, ordered by the frequency with which they appeared in the included studies. Figure 4 presents a compilation of the key variables by type identified in the studies from this review.

## 4 Discussion

This scoping review identified the main mediating and moderating variables, along with their respective instruments and samples, in studies on VTI among public servants working in the executive branch over the last 20 years. The findings provide a comprehensive overview based on the inclusion of 51 articles. There was an inclusion of studies from developing and Asian countries, such as China, the United Arab Emirates, Indonesia, and three African countries (Egypt, Ghana, and Nigeria). Unfortunately, studies conducted with Latin American populations were not included in this review. However, the reality in developing countries, particularly in Africa, is likely to approximate a cultural context similar to that of Brazil’s public service.

The growing number of studies over the years reinforces the increasing concern within public service regarding VTI. Research models are complex and seek to identify how various variables act collectively to influence cognitions about leaving. Only five variables were assessed as both moderators and mediators: person-organization fit, work engagement, organizational identification, distributive justice, and job satisfaction. This reinforces Hayes (2018) assertion that everything depends on how the variable is conceptualized, investigated, and operationalized.

Seven variables identified in the studies included in this review acted as full mediators of other relationships. These variables were: person-organization fit, goal clarity, intrinsic motivation, organizational prestige, leader-member exchange, job satisfaction, and organizational support. These seven variables caused the direct relationship to cease to exist, occurring only through the mediating variable (Hayes, 2018).

This raises the question of whether this effect might also occur in organizational contexts different from those of the original studies. The hypothesis of a moderated mediation effect—in which a third, as-yet-unidentified variable moderates the mediator—cannot be dismissed (Hayes, 2018). Similarly, another mediation effect may have occurred. Therefore, testing multiple mediation and moderation pathways within the same model opens new avenues of analysis in the pursuit of more accurate and robust effects (Hayes, 2018).

The findings of this scoping review indicate that variables found in the dual role of mediator or moderator can be explored to add explanatory variance regarding the effects on VTI. Examples of this role plasticity are evident in variables such as person-organization fit, job satisfaction, work engagement, distributive justice, job insecurity, and age. However, this can only be definitively validated by employing a longitudinal design that involves collecting multiple measures of the same variables with the same scale over time (Abbad and Carlotto, 2016).

The motivation, attitudes, and work-related behaviors category (Table 1) includes the variables job satisfaction, intrinsic motivation and public service motivation. Job satisfaction remains the most studied variable, primarily because early theories only linked this dichotomy to job dissatisfaction (Mobley et al., 1978; March and Simon, 1979). Classical models measure satisfaction as having a direct relationship with VTI and turnover, as if it were a pure cause-and-effect relationship—in other words, that a dissatisfied public employee will leave the organization.

Job satisfaction is viewed as the sum of an individual’s expectations regarding their work (Porter and Steers, 1973; Rubenstein et al., 2018). Studies have predominantly investigated its mediating role, i.e., its interference in direct relationships. The research in this review shows that job satisfaction is predominantly investigated for its mediating role—that is, its influence on direct relationships. In three studies, this variable was a full mediator in the relationships between VTI and: principal support, organizational learning culture, and person-organization fit (Liu et al., 2010; Lin et al., 2022; Al-Mahdy and Alazmi, 2023). In these studies, direct relationships disappeared in the presence of job satisfaction. In a study in South Africa, job satisfaction weakened the strength of the relationship between talent management practices and VTI (Barkhuizen and Gumede, 2021). This underscores that precisely operationalizing the variable, by tailoring it to the specific context where the assessment of VTI is required, yields more accurate results.

Intrinsic motivation results from an individual’s positive reaction to work and deals with performing an activity for the satisfactions and interest in the task itself (Deci et al., 2017). This variable fully mediated the direct relationship between ethical leadership and VTI in a study in Iraq (Shareef and Atan, 2019). Public service motivation appears to be an important variable for Asian and collectivist cultures. Public service motivation is conceptualized as an individual’s response to motives grounded in the public interest, a characteristic of public organizations (Perry, 1996; Perry et al., 2010). This variable has been investigated as a moderator that consistently weakens VTI. This variable moderated the relationship between VTI and: occupational stress, perceived overqualification, emphasis on efficiency, and work demands (Campbell et al., 2014; Shim et al., 2017; Bao and Zhong, 2021, 2023).

In Brazil, the concept of vocation in public service has been discussed, characterized by an orientation toward collective action, the prioritization of democratic values in decision-making, a commitment to equal rights, and the assurance of transparency (Coelho and Menon, 2018; Araújo et al., 2020; Gomide et al., 2023). Both intrinsic motivation and public service motivation can be utilized in empirical research to assess this vocation and the retention of public servants.

The following variables are part of the person-organizational context fit grouping (Table 2): person-organization fit, organizational identification, distributive justice, and leader-member exchange. Person-organization fit deals with the congruence between the individual and the organization in which they work (Kristof-Brown et al., 2005; Rubenstein et al., 2018). This variable appears as a moderator (Nguyen N. T. H. et al., 2023) and as a full mediator in two separate instances. These studies were conducted in countries with very different contexts—the USA and Pakistan—and analyzed very distinct fields, such as health and education (Bright, 2008; Kakar et al., 2019).

Organizational identification was a moderator in the relationship between VTI and organizational change (Boon et al., 2021). This variable acted as a partial mediator in the relationship between VTI and organizational stressors, with Chinese public servants (Bao and Zhong, 2021). Organizational identification deals with the degree to which public servants internalize the norms, values, and interests of the organization (Ashforth and Mael, 1989; Riketta, 2005).

Distributive justice deals with individuals’ perceptions of the outcomes or allocations received in relation to the work performed (Folger and Cropanzano, 2001; Colquitt, 2012). This variable appeared as a partial mediator of the relationship between talent management practices and VTI (Supi et al., 2023). Distributive justice was tested as a moderator in two studies (Campbell et al., 2014; Chordiya, 2022a). Chordiya’s (2022a) study showed that distributive justice weakened the strength of VTI for public servants who identified as non-white.

There is limited research in the public sector on how racial differences may influence VTI. No studies addressing this topic were found for Asian, African, or Latin American countries. In Brazil, for instance, discrimination is often subtle, masked by the discourse of racial mixing (Matos, 2018; Santos and Sales, 2018). Replications of Chordiya’s (2022a) study in Asian, African, and Latin American countries could help reveal these differences and determine the importance of distributive justice.

The relationship between information sharing and VTI was also fully suppressed by the presence of leader-member exchange (Ertürk, 2014). Leader-member exchange refers to the vertical relationship between a leader and the members of a work team (Graen and Uhl-Bien, 1995). Research has linked high-quality leader-member relationships to the emergence of innovative behavior (Ng, 2017), suggesting that environments that foster innovation and creativity might mitigate VTI.

Full mediating effects were found for goal clarity (Caillier, 2016b), organizational prestige (Bright, 2021), and POS (Ertürk, 2014), variables from the organizational context grouping (Table 3). The relationship between VTI and transformational leadership ceased to exist in the presence of goal clarity in research conducted in the USA (Caillier, 2016b). Goal clarity refers to the extent to which objectives and goals are clearly defined for employees (Sawyer, 1992). Also in a U.S. study, the relationship between public service motivation and VTI occurred only through the full mediation of perceived organizational prestige (Bright, 2021). Perceived organizational prestige is employees’ belief about how their organization is viewed by others (Mael and Ashforth, 1992).

Perceived organizational support fully mediated the relationship between participation in decision-making, shared information, and fair rewards, and VTI (Ertürk, 2014). POS seeks to assess public servants’ beliefs about the extent to which the organization values their contributions and cares about their wellbeing (Eisenberger et al., 1986). Public service in countries such as Pakistan and Brazil is often plagued by allegations of corruption (Bueno et al., 2016; Arslan, 2018). The general public frequently associates the corruption found within a specific agency with the civil servants who work there, leading to a lower perceived prestige that may, in turn, increase VTI. Furthermore, such allegations obscure organizational objectives, as corruption hinders the achievement of planned goals. Research examining goal clarity, organizational prestige, and POS as mediators can help explore the broader organizational context in which VTI is embedded.

Work engagement appears in the personal conditions resulting from work category (Table 4). This variable refers to the level of involvement, commitment, and energy that public servants invest in the organization (Schaufeli and Bakker, 2004). In the study by Obuobisa-Darko and Sokro (2023), work engagement had a negative moderating effect, minimizing the positive effect of the psychological impact of the COVID-19 pandemic on VTI. In turn, the relationship between work-family conflict and VTI was partially mediated by work engagement in the study by Yucel et al. (2023). Both studies were conducted in healthcare contexts, the first in an African country, Ghana, and the other in an Asian country, Turkey. Both studies based their assumptions on the Job Demands-Resources theory (Demerouti et al., 2001).

The human resource management practices grouping (Table 5) shows no repetition of any variable. The studies analyzed variables linked to the most current discussions related to inclusion, diversity, and flexible work arrangements. Pro-diversity management positively moderated the relationship between racial identity and VTI (Chordiya, 2022a). One study analyzed the moderating effects of agency type and inclusive practices on the relationship between belonging to the lesbian, gay, bisexual, and transgender (LGBT) public servant population and VTI. The moderating effect was observed when fair inclusive practices were perceived and also when agency types were redistributive (female-dominated) or regulatory (Sabharwal et al., 2019).

Flexible work arrangements mitigated the effects of the relationship between family responsibility discrimination and VTI in Canadian public servants. Mitigating effects were found for compressed workweeks, while no differences were observed for those engaged in telework or utilizing flextime (Mullins et al., 2021). Considering that these more flexible work arrangements were widely adopted during the COVID-19 pandemic and have been maintained in many organizations even after the abatement of the health crisis, further investigation into these moderating effects in other countries and cultural contexts is important (Lucas and Santos, 2021; Oliveira and Pantoja, 2021; Vilarinho et al., 2021; Carvalho et al., 2023).

Studies on inclusion, diversity, and flexible work arrangements have been conducted since 2021, following the pandemic period. Diverse and inclusive environments are hallmarks of human rights in the public sector, embedded in the concept of equality, a principle present in the constitutions of democratic countries such as Brazil and Chile (Bittencourt Friedrich and Gesta Leal, 2015; Bugueño, 2017). There is still very little research on VTI in these specific minority populations, people with disabilities, indigenous and LGBT (Sabharwal et al., 2019; Chordiya, 2022a,b). In Brazil, inequalities in both gender and race within the federal public service are under discussion (Faria, 2016; Alencar, 2021; Penha and Picanço, 2022).

The Covid-19 pandemic led to the abrupt implementation of remote work worldwide. In the case of public servants, the work is performed outside their organization’s premises through the use of technological tools. Even today, remote work remains in place in many areas of the federal public service (Lucas and Santos, 2021; Oliveira and Pantoja, 2021). No studies analyzing the relationship between voluntary turnover intention (VTI) and remote work in Brazil have been found.

Regarding demographic characteristics (Table 6), a study comparing work motivation variables with VTI found that belonging to the millennial generation moderated the relationship only for the variables of creativity and job satisfaction, which proved more significant for millennials than for the older generation (Ertas, 2015). The interaction between age and career opportunities attenuated turnover intention (IVR) for older workers (Pitts et al., 2011). Employees with greater autonomy experienced a greater reduction in VTI than their younger counterparts (Senapaty and Venugopal, 2023). Regarding the results, younger public servants showed a higher probability of VTI in all studies.

Brazil is facing an aging public service workforce within its federal executive branch, where the current average age is 47 (Brasil, 2023). This trend mirrors the broader demographic shift in the country, as the elderly population increased from 8 million in 1980 to 30 million in 2020 (Adamczyk, 2021). Consequently, a 2024 civil service examination aimed to fill vacant positions by hiring individuals in their 30’s (Brasil, 2025). The same work environment will be composed of civil servants from different generations: Generation X (born 1965–1980), Generation Y or Millennials (born 1981–1996), and Generation Z (born after 1997). Some studies have found support for generational differences based on individuals’ distinct and shared social experiences (Wey Smola and Sutton, 2002; Parry and Urwin, 2011; Rani and Samuel, 2016). Given that current work environments involve different generations coexisting and working together, analyzing the effects of age (generational cohort) on VTI in other organizational contexts and countries could be insightful.

In the meta-analysis by Hur and Abner (2024), which focuses on the public sector, the following appear as antecedents: (a) with a high effect size: job satisfaction, and (b) with a moderate effect size: intrinsic motivation. The meta-analysis by Rubenstein et al. (2018) identifies the following antecedents: (a) with a moderate effect size: person-organization fit and job satisfaction; (b) with a weak effect size: work engagement, justice (aggregating all subtypes), organizational prestige, and organizational support.

The different mediators and moderators presented in this review demonstrate that the public sector is increasingly adopting more complex analyses, moving beyond simple cause-and-effect relationships between an antecedent or predictor variable and the variable of interest. The pathway of VTI and turnover involves models with multiple mediators and moderators, including moderated mediation or mediated moderation analyses, employing more sophisticated statistical methods such as path analysis or structural equation modeling (Hayes, 2018; Hair et al., 2019).

## 5 Limitations and future research

The primary limitation of this research pertains to the number of databases investigated; therefore, other articles may exist that were not included in the present analysis. However, it is assumed that the databases utilized encompass the majority of relevant indexed publications. The adopted search strategy made it possible to include a broader range of Asian and developing countries, which were absent in other studies. Future studies could focus, for example, on literature from Central and South America, from which, despite efforts, no studies were included in this review.

Another limitation is the heterogeneous nature of the variables identified, which precludes more advanced quantitative analysis through methods such as statistical clustering. For instance, the variable “satisfaction,” present in 11 studies, was measured using different methods in each. The non-comparability of scales, measurement methods, and sample selection procedures complicates a quantitative approach to the findings, which could have aided in their interpretation. Nevertheless, our data synthesis focused on aggregation into six distinct categories, aiming to provide an overview, particularly of the variables that most frequently appear in research as mediators and moderators, an aspect not presented in previous reviews.

Existing studies are scarce and largely limited to the analysis of direct relationships, often overlooking complex scenarios where multiple variables are measured to assess their effects on VTI and turnover. This research gap has been specifically identified in the Brazilian context by two recent literature reviews (Beria et al., 2018; Seidl et al., 2019). The use of path analysis and structural equation modeling to evaluate sequential or hierarchical mediations, combined with the influence of moderators, should be employed to determine the true effects of variables on the outcome variable (VTI) and turnover (Hayes, 2018; Hair et al., 2019).

Future studies should aim to include moderating variables related to inclusion, diversity, and flexible work arrangements, discussions of which are highly prevalent in discourse concerning the post-pandemic work environment. Future research could primarily incorporate variables that demonstrated total mediating effects, such as goal clarity, intrinsic motivation, organizational prestige, job satisfaction, leader-member exchange, POS, and person-organization fit, due to their effect of negating a direct relationship with VTI.

Future studies also need to consider longitudinal assessments using these variables to effectively evaluate over time how this process leading to VTI and turnover unfolds. The number of studies that employed such a design was negligible. Future research should seek to assess specific mediators and moderators using validated scales, applying them in different contexts with time-lagged measurements to evaluate, through a longitudinal panel, whether such characteristics persist over time.

## 6 Conclusion

This review contributes to the literature on voluntary turnover intention (VTI) by presenting the main mediators and moderators studied in the public sector. The identified variables can support future research focused on specific subgroups of public servants and on better-defined strategies for diagnosing and monitoring VTI. Fifteen studies included only moderating variables, 26 studies assessed mediating relationships, and 10 assessed both, addressing gaps highlighted in two previous reviews (Hur and Abner, 2024; Rubenstein et al., 2018). These variables were grouped into six different dimensions.

There remains, however, a lack of representation of studies in underdeveloped countries, especially as no eligible studies were found in Central and South America. The significant number of variables identified demonstrates the growing concern with tailoring research and identifying what appears to be most relevant in real-world contexts. Furthermore, a selection of variables was presented that, due to their full mediating effect or their role as moderators, may represent important stages in the pathway leading to VTI.

Human Resources (HR) departments within public sector organizations can leverage the key mediating and moderating variables from this review to periodically assess employee turnover and VTI. For instance, conducting annual assessments would allow them to track the behavior of these variables over time, providing a clear diagnosis of emerging trends. With this diagnosis, these departments can then develop and implement targeted action plans to mitigate the negative influence of specific factors on both turnover and VTI.

## Data Availability

Publicly available datasets were analyzed in this study. This data can be found here: https://osf.io/eb3uf/.
