# Supplementary material for: Mediators and moderators in voluntary turnover intention: a scoping review in the public service
Source: Front Psychol. 2025 Oct 28;16:1631551. doi: 10.3389/fpsyg.2025.1631551 (PMC12604477; doi:10.3389/fpsyg.2025.1631551)
Supplement: Supplementary file 1 [file Data_Sheet_1.pdf]

Appendix A
Moderating and mediating variables and main characteristics of included studies

					Instrument		Country		Study Characteristics		Sample	
Variables	Year	Author	Type	Scale	Measurement method	Sample item	Author	Study	Design	Sample size	Type	Work Area
Job satisfaction	2021	Barkhuizen & Gumede	Mod	10 items developed for the research.	Likert-type (1 = strongly disagree; 6 = strongly agree).	Items not presented.	South Africa	South Africa	CS	201-300	R	Agencies
Job satisfaction	2023	Al-Mahdy & Alazm	Med	5 items (Ho & Au, 2006)	Likert-type (1 = strongly disagree; 5 = strongly agree).	I am satisfied with being a teacher.	Egypt and Kuwait	Kuwait	CS	301-400	C	Education
Job satisfaction	2010	Liu et al.	Med	4 items (B. Liu et al., 2008)	Likert-type (1 = strongly disagree; 6 = strongly agree).	Most days I am enthusiastic about my work.	China	China	CS	201-300	C	Multiple areas
Job satisfaction	2018	Jabeen et al.	Med	5 items (Judge et al., 2000).	Likert-type (1 = strongly disagree; 7 = strongly agree).	Most days I am enthusiastic about my work.	United Arab Emirates	United Arab Emirates	CS	301-400	R	Multiple areas
Job satisfaction	2018	Jin et al.	Med	4 items adapted from (Corley & Sabharwal, 2007)	Likert-type (1 = very dissatisfied; 5 = very satisfied)	Thinking about your main job, rate your satisfaction with the intellectual challenge	USA and Republic of Korea	USA	CS	acima de 500	C	Education

					Instrument		Country		Study Characteristics		Sample	
Variables	Year	Author	Type	Scale	Measurement method	Sample item	Author	Study	Design	Sample size	Type	Work Area
Job satisfaction	2017	S. Y. Kim & Fernandez	Med	2 items developed for the research.	Likert-type (1 = strongly disagree; 5 = strongly agree).	I like the type of work I do	USA	USA	CS	acima de 500	R	Agencies
Job satisfaction	2022	C.-Y. Lin et al.	Med	Michigan Organizational Assessment Questionnaire (Cammann et al., 1983)	Likert-type (1 = strongly disagree; 5 = strongly agree).	Overall, I enjoy working here	Thailand	Thailand	CS	301-400	C	Multiple areas
Job satisfaction	2023	Y. Liu et al.	Med	4 items (Judge et al., 1998)	1 = extremely inconsistent to 5 = extremely consistent	I feel quite satisfied with my current job	China	China	CS	acima de 500	C	Education
Job satisfaction	2024	Wang et al.	Med	4 items (Boateng & Hsieh, 2019).	Likert-type (1 = strongly disagree; 5 = strongly agree).	Overall, I am satisfied with my current job	Thailand and China	China	CS	acima de 500	C	Multiple areas
Job satisfaction	2024	Wesemann	Med	City of Tempe, Arizona, Biennial Survey	Likert-type (1 = very dissatisfied; 4 = very satisfied)	Overall, how satisfied are you with your current job?	USA	USA	CS	acima de 500	R	Multiple areas

					Instrument		Country		Study Characteristics		Sample	
Variables	Year	Author	Type	Scale	Measurement method	Sample item	Author	Study	Design	Sample size	Type	Work Area
Job satisfaction	2023	Zhang et al.	Med	5 items developed for the research.	Likert-type (1 = very dissatisfied; 5 = very satisfied)	Overall, are you satisfied with your current job as a teacher?	China	China	CS	201-300	R	Education
Burnout	2023	Aman-Ullah et al.	Med	10 items adapted from (Maslach et al., 2001)	Likert-type (1 = strongly disagree; 7 = strongly agree).	I feel emotionally exhausted by my work	Malaysia, Saudi Arabia, Spain, and Bangladesh	Pakistan	CS	201-300	C	Health
Burnout	2015	J. Kim	Med	22 items (Maslach & Jackson, 1981)	Likert-type (1 = strongly disagree; 7 = strongly agree).	I feel drained at the end of the workday	Republic of Korea	Republic of Korea	CS	201-300	R	Inspection /Regulation
Burnout	2021	Samad et al.	Med	10 items Burnout Measure Short (Pines & Aronson, 1988).	Likert-type (1 = strongly disagree; 7 = strongly agree).	I feel emotionally drained by my work	Pakistan	Pakistan	CS	201-300	R	Health
Burnout	2017	Shim et al.	Med	National Survey of the Ministry of Public Administration and Security	Likert-type (1 = strongly disagree; 7 = strongly agree).	I feel drained at the end of the workday	Republic of Korea	Republic of Korea	CS	acima de 500	R	Others

				Instrument			Country		Study Characteristics		Sample	
Variables	Year	Author	Type	Scale	Measurement method	Sample item	Author	Study	Design	Sample size	Type	Work Area
Work engagement	2023	Obuobisa-Darko & Sokro	Mod	17 items (Schaufeli et al., 2002)	Likert-type (1 = strongly disagree; 5 = strongly agree).	I feel full of energy at work	Ghana	Ghana	CS	101-200	C	Multiple areas
Work engagement	2021	Bas & Çınar	Med	9 items adapted from (Anadolu University et al., 2015; Schaufeli & Bakker, 2004)	Likert-type (1 = strongly disagree; 5 = strongly agree).	I feel happy when I am working intensely	Turkey	Turkey	CS	401-500	R	Health
Work engagement	2023	Yucel et al.	Med	9 items adapted from (Anadolu University et al., 2015; Schaufeli & Bakker, 2004	Likert-type (1 = strongly disagree; 5 = strongly agree).	I feel happy when I am working intensely	Turkey	Turkey	CS	301-400	R	Health
Work engagement	2023	Diko & Saxena	Med	17 items (Schaufeli et al., 2006)	Likert-type (1 = Never; 5 = Always)	At my job, I feel strong and vigorous	India and Ethiopia	Ethiopia	CS	401-500	R	Education
Work engagement	2020	Gadi & Kee	Med	9 items (Schaufeli et al., 2006)	Likert-type (1 = strongly disagree; 5 = strongly agree).	I feel happy when I am working intensely	Malaysia and Nigeria	Nigeria	CS	301-400	C	Education

				Instrument			Country		Study Characteristics		Sample	
Variables	Year	Author	Type	Scale	Measurement method	Sample item	Author	Study	Design	Sample size	Type	Work Area
Public service motivation	2021	Bao & Zhong	Mod	4 items (Wright et al., 2013)	Likert-type (1 = strongly disagree; 5 = strongly agree).	Meaningful public service is very important to me	China	China	CS	301-400	C	Not described
Public service motivation	2023	Bao & Zhong	Mod	5 items (Wright et al., 2013)	Likert-type (1 = strongly disagree; 5 = strongly agree).	Meaningful public service is very important to me	China	China	CS	401-500	C	Multiple areas
Public service motivation	2014	Campbell et al.	Mod	5 items (Perry, 1997)	Likert-type (1 = strongly disagree; 5 = strongly agree).	I feel a strong responsibility towards society	Republic of Korea	Republic of Korea	CS	acima de 500	R	Multiple areas
Public service motivation	2017	Shim et al.	Mod	3 items (Perry, 1996)	Likert-type (1 = strongly disagree; 7 = strongly agree).	Meaningful public service is very important to me	Republic of Korea	Republic of Korea	CS	acima de 500	R	Others
Distributive justice	2014	Campbell et al.	Mod	1 item developed for the research.	Likert-type (1 = strongly disagree; 5 = strongly agree).	Promotion depends on employee performance	Republic of Korea	Republic of Korea	CS	acima de 500	R	Multiple areas

				Instrument			Country		Study Characteristics		Sample	
Variables	Year	Author	Type	Scale	Measurement method	Sample item	Author	Study	Design	Sample size	Type	Work Area
Distributive justice	2022	Chordiya	Mod	Federal Employee Viewpoint Survey - FEVS (2006-2017)	Likert-type (1 = strongly disagree; 5 = strongly agree).	My performance appraisal is a fair reflection of my performance	USA	USA	L	acima de 500	R	Multiple areas
Distributive justice	2023	Supi et al.	Med	3 items (Gelens et al., 2015; Loi et al., 2009)	Likert-type (1 = strongly disagree; 5 = strongly agree).	I feel that the award I received reflected the effort I have been making	Indonesia	Indonesia	CS	301-400	R	Inspection /Regulation
Perceived organizational support	2014	Ertürk	Med	6 items (Eisenberger et al., 1986). Ex.:	Likert-type (1 = strongly disagree; 5 = strongly agree).	My organization cares about my opinions	Turkey	Turkey	CS	201-300	R	Others
Perceived organizational support	2023	Y. Liu et al.	Med	6 items (Eisenberger et al., 1986; Loi et al., 2006)	1 = extremely inconsistent to 5 = extremely consistent	My organization cares about my opinions	China	China	CS	acima de 500	C	Education
Perceived organizational support	2023	Supi et al.	Med	8 items (Eisenberger et al., 2002; Gelens et al., 2014)	Likert-type (1 = strongly disagree; 5 = strongly agree).	The organization appreciates employees' extra effort	Indonesia	Indonesia	CS	301-400	R	Inspection /Regulation

				Instrument			Country		Study Characteristics		Sample	
Variables	Year	Author	Type	Scale	Measurement method	Sample item	Author	Study	Design	Sample size	Type	Work Area
Person-organization fit	2023	N. T. H. Nguyen et al.	Mod	4 items (Wright & Pandey, 2008)	Likert-type (1 = strongly disagree; 7 = strongly agree).	My personal values match the organization's values	United Kingdom, Vietnam, and Australia	USA	CS	401-500	C	Multiple areas
Person-organization fit	2008	Bright	Med	4 items (Kristof-Brown et al.,2005)	Likert-type (1 = strongly disagree; 7 = strongly agree).	My values and goals are very similar to my organization's values and goals	USA	USA	CS	201-300	R	Health
Person-organization fit	2019	Kakar et al.	Med	5 items (Vogel & Feldman, 2009)	Likert-type (1 = strongly disagree; 5 = strongly agree).	The things I value in life are very similar to the things my institution values	Malaysia	Pakistan	CS	201-300	C	Education
Age	2011	Pitts et al.	Mod	Polytomous	30, 30–39, 40–49, 50–59, Over 60	Not presented	USA	USA	CS	acima de 500	R	Multiple areas
Age	2023	Senapaty & Venugopal	Mod	Dichotomous	1 (under 20 years) to 7 (over 70 years)	Not presented	India	India	CS	301-400	C	Others
Job insecurity	2023	Wirawan et al.	Mod	4 items (Vander Elst et al., 2014)	Likert-type (1 = strongly disagree; 5 = strongly agree).	I feel insecure about the future of my job	Indonesia	Indonesia	CS	201-300	C	Multiple areas

				Instrument			Country		Study Characteristics		Sample	
Variables	Year	Author	Type	Scale	Measurement method	Sample item	Author	Study	Design	Sample size	Type	Work Area
Job insecurity	2023	Kakar et al.	Med	5 items adapted from (De Witte, 2000)	Likert-type (1 = strongly disagree; 5 = strongly agree).	I feel I might lose my job because of the COVID-19 pandemic	Pakistan, Malaysia, and South Africa	Pakistan	CS	201-300	C	Education
Procedural justice	2014	Campbell et al.	Mod	2 items (Thibaut & Walker, 1975) e 1 item developed for the research.	Likert-type (1 = strongly disagree; 5 = strongly agree).	Employees are involved in decisions that affect them	Republic of Korea	Republic of Korea	CS	acima de 500	R	Multiple areas
Procedural justice	2022	Chordiya	Mod	FEVS (2006-2017)	Likert-type (1 = strongly disagree; 5 = strongly agree).	I can report a suspected violation of any law, rule, or regulation without fear of reprisal	USA	USA	L	acima de 500	R	Multiple areas
Perceived supervisor support	2023	Yucel et al.	Mod	7 items (Gant, et al., 1993)	Likert-type (1 = strongly disagree; 5 = strongly agree).	My manager helps get things done	Turkey	Turkey	CS	301-400	R	Health
Perceived supervisor support	2013	Dysvik & Kuvaas	Mod	4 items (Eisenberger et al., 1986)	Likert-type (1 = strongly disagree; 5 = strongly agree).	My work supervisor really cares about my well-being	Norway	Norway	CS	acima de 500	C	Multiple areas

				Instrument			Country		Study Characteristics		Sample	
Variables	Year	Author	Type	Scale	Measurement method	Sample item	Author	Study	Design	Sample size	Type	Work Area
Organizational identification	2021	Boon et al.	Mod	Australian Public Servants Survey 2014	Likert-type (1 = strongly disagree; 5 = strongly agree).	I feel a strong personal attachment to my agency	Belgium	Australia	CS	acima de 500	R	Multiple areas
Organizational identification	2021	Bao & Zhong	Med	5 items (Smidts et al., 2001)	Likert-type (1 = strongly disagree; 7 = strongly agree).	I feel strong ties to my current organization	China	China	CS	301-400	C	Not described
Agency type	2019	Sabharwal et al.	Mod	Dichotomous	1 and 0	Redistributive, distributive, constituent, and regulatory	USA	USA	CS	acima de 500	R	Agencies
Benefits Package	2023	Xu et al.	Mod	Dichotomous	Yes and No	Do you receive it?	China	China	CS	acima de 500	C	Health
Career commitment	2022	Li & Xie	Med	6 items (Blau, 1989)	Likert-type (1 = strongly disagree; 5 = strongly agree).	This is the ideal career for a professional life	China	China	CS	301-400	C	Multiple areas
Career Growth Opportunities	2024	Wang et al.	Mod	3 items (Zhang & Zhang, 2007)	Likert-type (1 = strongly disagree; 5 = strongly agree).	My organization offers me opportunities to keep up with development trends related to my work	Thailand and China	China	CS	acima de 500	C	Multiple areas

				Instrument			Country		Study Characteristics		Sample	
Variables	Year	Author	Type	Scale	Measurement method	Sample item	Author	Study	Design	Sample size	Type	Work Area
Change-oriented organizational citizenship behavior	2016	Campbell & Im	Med	4 items (Morrison & Phelps, 1999)	1 = strong disagreement; 5 = strong agreement	I try to change work processes to increase efficiency	Republic of Korea and Rússia	Republic of Korea	CS	401-500	R	Others
Collaborative culture	2017	R. Sun & Wang	Med	Teacher Survey New York State Education Department.	1 = strongly disagree; 4 = strongly agree	The teachers at my school trust each other	USA	USA	CS	acima de 500	R	Education
Employee loyalty	2022	Albtoosh et al.	Med	4 items (Yee, 2010)	Likert-type (1 = strongly disagree; 7 = strongly agree).	I would accept any job to continue working for this company	Malaysia	Jordan	CS	301-400	C	Multiple areas
Ethical leadership	2023	T. D. Nguyen et al.	Mod	6 items (Brown et al., 2005)	Likert-type (1 = strongly disagree; 5 = strongly agree).	My boss listens to what employees have to say	Thailand and Vietnam	Vietnam	CS	201-300	C	Multiple areas
Flexible Work Arrangements	2021	Mullins et al.	Mod	Canadian Public Service Employee Survey (2002, 2005 e 2008)	Polytomous	Compressed workweek, flextime, and telework	USA and Canada	Canada	CS	acima de 500	R	Others

				Instrument			Country		Study Characteristics		Sample	
Variables	Year	Author	Type	Scale	Measurement method	Sample item	Author	Study	Design	Sample size	Type	Work Area
Followership behavior	2018	Jin et al.	Med	6 items (Blanchard et al., 2009)	Likert-type (1 = strongly disagree; 5 = strongly agree).	I take the initiative to seek out and successfully complete tasks that go beyond my job	USA and Republic of Korea	USA	CS	acima de 500	C	Education
Goal clarity	2016	Caillier	Med	10 items(Sawyer, 1992)	Likert-type (1 = strongly disagree; 7 = strongly agree).	I perform poorly when there is little communication in a work situation	USA	USA	CS	acima de 500	R	Multiple areas
Inclusion Quotient	2019	Sabharwal et al.	Mod	FEVS (2015)	1 = negative, 2 = neutral, and 3 = positive	In my work unit, performance differences are recognized in a meaningful way	USA	USA	CS	acima de 500	R	Agencies
Inclusive Organizational Practices	2022	Chordiya	Mod	FEVS (2012-2015)	Likert-type (1 = strongly disagree; 5 = strongly agree).	My performance appraisal is a fair reflection of my performance	USA	USA	CS	acima de 500	R	Multiple areas

				Instrument			Country		Study Characteristics		Sample	
Variables	Year	Author	Type	Scale	Measurement method	Sample item	Author	Study	Design	Sample size	Type	Work Area
Incivility	2023	Wirawan et al.	Mod	Cortina et al., 2001	Likert-type (0 = never; 4 = almost every day)	Have any of your superiors or colleagues belittled you or been condescending to you?	Indonesia	Indonesia	CS	201-300	C	Multiple areas
Intrinsic motivation	2019	Shareef & Atan	Med	5 items (Tierney et al., 1999)	Likert-type (1 = strongly disagree; 5 = strongly agree).	I enjoy finding solutions to complex problems	Cyprus	Iraq	CS	301-400	R	Education
Leader-member exchange	2014	Ertürk	Med	8 items de (Graen & Uhl-Bien, 1995)	Likert-type (1 = strongly disagree; 5 = strongly agree).	Do you think your immediate supervisor understands your problems and needs?	Turkey	Turkey	CS	201-300	R	Others
Life satisfaction	2023	T. D. Nguyen et al.	Med	5 items (Larsen et al., 1985)	Likert-type (1 = strongly disagree; 7 = strongly agree).	In many respects, my life is close to my ideal	Thailand and Vietnam	Vietnam	CS	201-300	C	Multiple areas
Millennial Status	2015	Ertas	Mod	Dichotomous	≥ 29 years e ≤ 29 years	Not presented	USA	USA	CS	acima de 500	R	Agencies

				Instrument			Country		Study Characteristics		Sample	
Variables	Year	Author	Type	Scale	Measurement method	Sample item	Author	Study	Design	Sample size	Type	Work Area
Mission valence	2016	Caillier	Med	3 items (Wright e Pandey, 2011)	Likert-type (1 = strongly disagree; 7 = strongly agree).	I believe my employing organization's priorities are very important	USA	USA	CS	acima de 500	R	Agencies
Negative affect	2023	Bao & Zhong	Med	5 items (B. Liu et al., 2008)	Likert-type (1 = Never; 5 = Always, extremely frequent)	My job makes me angry	China	China	CS	401-500	C	Multiple areas
Occupational stress	2021	Samad et al.	Med	10 items (Cohen et al., 1983)	Likert-type (1 = strongly disagree; 7 = strongly agree).	In the last month, how often have you been upset because of something that happened unexpectedly?	Pakistan	Pakistan	CS	201-300	R	Health
Off-the-job embeddednes	2018	Hussain & Deery	Mod	3 items (Felps et al., 2009)	Likert-type (1 = strongly disagree; 5 = strongly agree).	My family roots are in this community	United Arab Emirates	United Arab Emirates	CS	201-300	C	Health

					Instrument		Country		Study Characteristics		Sample	
Variables	Year	Author	Type	Scale	Measurement method	Sample item	Author	Study	Design	Sample size	Type	Work Area
Organizational climate for innovation	2014	Campbell et al.	Mod	4 items (Scott & Bruce, 1994)	Likert-type (1 = strongly disagree; 5 = strongly agree).	My organization's leadership encourages creative solutions to problems	Republic of Korea	Republic of Korea	CS	acima de 500	R	Multiple areas
Organizational trust	2014	Ertürk	Mod	4 items adapted from (Nyhan & Marlowe, 1997)	Likert-type (1 = strongly disagree; 5 = strongly agree).	I am sure that people in this organization trust each other	Turkey	Turkey	CS	201-300	R	Others
Perceived competence mobilization	2009	Lai & Kapstad	Med	10 items developed for the research.	Likert-type (1 = To a very small extent; 5 = To a great extent)	To what extent do you have the opportunity to use your knowledge, skills, and abilities in your current job?	Norway	Norway	CS	acima de 500	C	Public safety
Perceived organizational prestige	2021	Bright	Med	3 items (Mael & Ashforth, 1992)	Likert-type (1 = strongly disagree; 7 = strongly agree).	People in my community have a very positive opinion of my agency	USA	USA	CS	acima de 500	C	Others

				Instrument			Country		Study Characteristics		Sample	
Variables	Year	Author	Type	Scale	Measurement method	Sample item	Author	Study	Design	Sample size	Type	Work Area
Pro-diversity Management	2022	Chordiya	Mod	FEVS (2006-2017)	Likert-type (1 = strongly disagree; 5 = strongly agree).	My supervisor/team leader is committed to a workforce representative of all segments of society	USA	USA	L	acima de 500	R	Multiple areas
Psychological distress	2023	N. T. H. Nguyen et al.	Med	10 items (Kessler et al., 2002)	Likert-type (1 = none of the time; 5 = all of the time)	In the last 30 days, have you felt agitated or restless?	United Kingdom, Vietnam, and Australia	USA	CS	401-500	R	Multiple areas
Self-efficacy	2016	Caillier	Med	5 items de Riggs et al. (1994)	Likert-type (1 = strongly disagree; 7 = strongly agree).	I am confident in my ability to do my job	USA	USA	CS	acima de 500	R	Multiple areas
Servant leadership	2022	Li & Xie	Mod	7 items (Liden et al., 2015)	Likert-type (1 = strongly disagree; 5 = strongly agree).	My leader puts my best interests ahead of their own	China	China	CS	301-400	C	Multiple areas
Training satisfaction	2022	Albtoosh et al.	Med	3 items (Sanduvete-Chaves et al., 2013)	Likert-type (1 = strongly disagree; 7 = strongly agree).	In my opinion, the planned objectives were achieved	Malaysia	Jordan	CS	301-400	C	Multiple areas

				Instrument			Country		Study Characteristics		Sample	
Variables	Year	Author	Type	Scale	Measurement method	Sample item	Author	Study	Design	Sample size	Type	Work Area
Trust in supervisor	2014	Ertürk	Mod	8 items adapted from (Nyhan & Marlowe, 1997)	Likert-type (1 = strongly disagree; 5 = strongly agree).	I trust my supervisor; I can rely on what he or she says	Turkey	Turkey	CS	201-300	R	Others
Turnover system type	2008	Strolin-Goltzman et al.	Med	Dichotomous	Low and High	Not presented	USA	USA	CS	acima de 500	C	Others

Legenda: Mod - Moderator / Med - Mediator / L - Longitudinal/ T - Transversal / C - Conveniência/ A – Aleatória

Appendix B
Articles Excluded in Full Reading

**Publication Year**	**Author**	**Title**	**Publication Title**	**Mediator and / or Moderator**	**Sample with active public servants in the executive branch?**	**VTI**	**Impossibility of the Article**
2004	Bartlett, Kenneth R.; McKinney, William R.	A study of the role of professional development, job attitudes, and turnover among public park and recreation employees.	Journal of Park & Recreation Administration	No			
2005	Kim, SH	Factors affecting state government information technology employee turnover intentions	American Review of Public Administration	No			
2005	Parker, RJ; Kohlmeyer, JM	Organizational justice and turnover in public accounting firms: A research note	Accounting Organizations And Society		No		
2005	Hart, Sara Elizabeth	Hospital ethical climates and registered nurses' turnover intentions.	Journal of nursing scholarship: An official publication of Sigma Theta Tau International Honor Society of Nursing	No			
2006	Daly, CJ; Dee, JR	Greener pastures: Faculty turnover intent in urban public universities	Journal of Higher Education			No	
2007	Tung-Chun, Huang; Lawler, J; Ching-Yi, L	The effects of quality of work life on commitment and turnover intention	Social Behavior and Personality		No		
2008	Moynihan, DP; Pandey, SK	The ties that bind: Social networks, person-organization value fit, and turnover intention	Journal of Public Administration Research And Theory		No		
2008	Moynihan, DP; Landuyt, N	Explaining turnover intention in state government examining the roles of gender, life cycle, and loyalty	Review of Public Personnel Administration	No			

Publication Year	Author	Title	Publication Title	Mediator and / or Moderator	Sample with active public servants in the executive branch?	VTI	Impossibility of the Article
2010	Jung, CS	Predicting organizational actual turnover rates in the U.S. federal government	International Public Management Journal	No			
2010	Ozer, G; Gunluk, M	The effects of discrimination perception and job satisfaction on Turkish public accountants' turnover intention	African Journal of Business Management		No		
2011	Lee, G; Jimenez, BS	Does performance management affect job turnover intention in the federal government?	American Review of Public Administration	No			
2012	Bradley, S; Green, C; Mangan, J	The effect of relative wages and external shocks on public sector turnover	Economic Record			No	
2012	Jerneic, Z; Kutlesa, V	Job attitudes, job performance and turnover intentions of scientists	Suvremena Psihologija				Yes
2012	Herda, DN; Lavelle, JJ	The auditor-audit firm relationship and its effect on burnout and turnover intention	Accounting Horizons				Yes
2013	Nouri, H; Parker, RJ	Career growth opportunities and employee turnover intentions in public accounting firms	British Accounting Review		No		
2013	Olusegun, Oyetola Solomon	Influence of job satisfaction on turnover intentions of library personnel in selected universities in South West Nigeria.	Library Philosophy & Practice	No			
2014	Jung, CS	Why are goals important in the public sector? Exploring the benefits of goal clarity for reducing turnover intention	Journal of Public Administration Research and Theory	No			

Publication Year	Author	Title	Publication Title	Mediator and / or Moderator	Sample with active public servants in the executive branch?	VTI	Impossibility of the Article
2014	Watty-Benjamin, W; Udechukwu, I	The relationship between HRM practices and turnover intentions: a study of government and employee organizational citizenship behavior in the Virgin Islands	Public Personnel Management	No			
2014	Asegid, Agezegn; Belachew, Tefera; Yimam, Ebrahim	Factors influencing job satisfaction and anticipated turnover among nurses in Sidama Zone public health facilities, South Ethiopia.	Nursing research and practice	No			
2015	Chong, VK; Monroe, GS	The impact of the antecedents and consequences of job burnout on junior accountants' turnover intentions: a structural equation modelling approach	Accounting and Finance		No		
2015	Chaudhary, Swati; Chaudhari, Suman	Relationship between psychological capital, job satisfaction and turnover intention of bank employees.	Indian Journal of Health & Wellbeing	No			
2015	Ayalew, Firew; Kols, Adrienne; Kim, Young-Mi; Schuster, Anne; Emerson, Mark R.; Roosmalen, J. van; Stekelenburg, Jelle; Woldemariam, Damtew; Gibson, Hannah	Factors affecting turnover intention among nurses in Ethiopia.	World health & population	No			
2016	Cohen, G; Blake, RS; Goodman, D	Does turnover intention matter? Evaluating the usefulness of turnover intention rate as a predictor of actual turnover rate	Review of Public Personnel Administration	No			

Publication Year	Author	Title	Publication Title	Mediator and / or Moderator	Sample with active public servants in the executive branch?	VTI	Impossibility of the Article
2016	Iguchi, Aya	[Job demand and job resources related to the turnover intention of public health nurses: An analysis using a job demands-resources model].	[Nihon koshu eisei zasshi] Japanese journal of public health				Yes
2017	George, J; Wallio, S	Organizational justice and millennial turnover in public accounting	Employee Relations	No			
2017	Jung, CS; Chan, HS; Hsieh, CW	Public employees' psychological climates and turnover intention: evidence from Korean central government agencies	Public Management Review	No			
2017	Gevrek, D; Spencer, M; Hudgins, D; Chambers, V	I can't get no satisfaction The power of perceived differences in employee intended retention and turnover	Personnel Review	No			
2018	Ali, SB; Bishu, S; Alkadry, M	Why men and women want to leave? Turnover intent among public procurement officers	American Review of Public Administration	No			
2018	Park, SM; Joaquin, ME; Min, KR; Ugaddan, RG	Do reform values matter? Federal worker satisfaction and turnover intention at the dawn of the trump presidency	American Review of Public Administration	No			
2018	Anjum, Ambreen; Muazzam, Amina	Workplace bullying and turnover intention among university teachers.	Journal of Arts & Social Sciences	No			
2019	Hayes, MS; Stazyk, EC	Mission congruence: to agree or not to agree, and its implications for public employee turnover	Public Personnel Management			No	
2019	Ajayi, SO; Olatunji, OA	Turnover causation amongst high school teachers in Nigeria	Africa Education Review	No			

Publication Year	Author	Title	Publication Title	Mediator and / or Moderator	Sample with active public servants in the executive branch?	VTI	Impossibility of the Article
2019	Jiang, HY; Wang, YA; Chui, E; Xu, YB	Professional identity and turnover intentions of social workers in Beijing, China: The roles of job satisfaction and agency type	International Social Work		No		
2019	Yu, HH	Work-life balance: an exploratory analysis of family-friendly policies for reducing turnover intentions among women in U.S. federal law enforcement	International Journal of Public Administration	No			
2020	Smith, KJ; Emerson, DJ; Boster, CR; Everly, GS	Resilience as a coping strategy for reducing auditor turnover intentions	Accounting Research Journal		No		
2020	Oruh, ES; Mordi, C; Ajonbadi, A; Mojeed-Sanni, B; Nwagbara, U; Rahman, M	Investigating the relationship between managerialist employment relations and employee turnover intention The case of Nigeria	Employee Relations	No			
2020	Mahlasela, S; Chinyamurindi, WT	Technology-related factors and their influence on turnover intentions: A case of government employees in South Africa	Electronic Journal of Information Systems in Developing Countries	No			
2020	Masenya, J; Ngoepe, M; Jiyane, V	Determinants of turnover intentions of librarians at the City of Johannesburg libraries in Gauteng province, South Africa	South African Journal of Libraries And Information Science	No			
2021	Sallaberry, JD; dos Santos, EA; Bortoluzzi, DA; Lunkes, RJ	Profile characteristics of public ministry servers and their relationship with the intent to turnover	Administracao Publica e Gestao Social	No			

Publication Year	Author	Title	Publication Title	Mediator and / or Moderator	Sample with active public servants in the executive branch?	VTI	Impossibility of the Article
2021	Kamau, O; Muathe, SMA; Wainaina, L	Teachers' turnover intentions: role of HRM practices in public secondary schools in Kenya	Cogent Business & Management	No			
2021	Pham, CD; Hoang, TPD; Nguyen, YT	Impact of work motivation on satisfaction and turnover of public universities lecturers	Journal of Asian Finance Economics and Business	No			
2021	Zhan, Y. W.; Feng, A. P.; Liang, B. W.; Gao, Y. X.; Lu, Y.; Wang, Z. Y.; Shu, Y. L.; Zou, H. C.	[Employment, turnover intention and the associated factors among public health graduates in China].	Zhonghua yu fang yi xue za zhi [Chinese journal of preventive medicine]				Yes
2022	Alkarabsheh, OHM; Jaaffar, AH; Fong, PW; Almaaitah, DAA; Alkharabsheh, ZHM	The relationship between leadership style and turnover intention of nurses in the public hospitals of Jordan	Cogent Business & Management	No			
2022	Hattab, S; Wirawan, H; Salam, R; Daswati, D; Niswaty, R	The effect of toxic leadership on turnover intention and counterproductive work behaviour in Indonesia public organisations	International Journal of Public Sector Management			No	
2022	Pinho, APM; da Silva, CRM; de Oliveira, ERD	Determinants of turnover intention in the public sector: a study in a federal educational institution	Administracao Publica e Gestao Social	No			
2022	Yusoff, NA; Yusliza, MY; Saputra, J	The linkages between procedural justice and job embeddedness on turnover intention: moderating role of personality	Polish Journal of Management Studies	No			

Publication Year	Author	Title	Publication Title	Mediator and / or Moderator	Sample with active public servants in the executive branch?	VTI	Impossibility of the Article
2022	Burakova, M; McDowall, A; Bianvet, C	Are organisational politics responsible for turnover intention in French Firefighters?	European Review of Applied Psychology-Revue Europeenne de Psychologie Appliquee		No		
2022	Wynen, J; Boon, J; Verlinden, S	Reform stress in the public sector? Linking change diversity to turnover intentions and presenteeism among civil servants using a matching approach	Public Performance & Management Review	No			
2022	Mercado, Micaela; Wachter, Karin; Schuster, Roseanne C.; Mathis, Cherra M.; Johnson, Emma; Davis, Olga Idriss; Johnson-Agbakwu, Crista E.	A cross-sectional analysis of factors associated with stress, burnout and turnover intention among healthcare workers during the COVID-19 pandemic in the United States.	Health & Social Care in the Community	No			
2022	Sun, Xiaoting; Zhang, Mengmeng; Lu, Zhanghong; Zhang, Zhaoyu; Zheng, Jialin Charlie; Cheng, Liming; Zeng, Lianhua; Qian, Yingli; Huang, Lei	Turnover intention and related factors among resident physicians in China under the standardised residency training programme: a cross-sectional survey.	BMJ open		No		

Publication Year	Author	Title	Publication Title	Mediator and / or Moderator	Sample with active public servants in the executive branch?	VTI	Impossibility of the Article
2022	Lotfi, Mojgan; Akhuleh, Omid Zadi; Judi, Aysan; Khodayari, Mohammadtaghi	Turnover intention among operating room nurses during the COVID-19 outbreak and its association with perceived safety climate.	Perioperative care and operating room management	No			
2023	Suzuki, K; Hur, H	Politicization, bureaucratic closedness in personnel policy, and turnover intention	Governance - An International Journal of Policy Administration and Institutions	No			
2023	Calhoun, M; McCarthy, V	An unintended consequence of flexible work arrangements (FWAs) - the role of peer resentment on turnover intentions in public accounting	Journal of Accounting and Organizational Change		No		
2023	dos Santos, EA; Sallaberry, JD; Bortoluzzi, DA; Flach, L; Saraiva, MCCM	Person-environment fit and turnover intention: a study with civil servants	Contabilidade Gestao e Governanca		No		
2023	Masenya, J	Relationship between organisational determinants and turnover intentions of librarians in public libraries in Gauteng province	South African Journal of Libraries And Information Science	No			
2023	Kaufmann, W; Borry, EL; DeHart-Davis, L	Can effective organizational rules keep employees from leaving? A study of green tape and turnover intention	Public Management Review	No			

Publication Year	Author	Title	Publication Title	Mediator and / or Moderator	Sample with active public servants in the executive branch?	VTI	Impossibility of the Article
2023	Badar, K; Aboramadan, M; Plimmer, G	Despotic vs narcissistic leadership: differences in their relationship to emotional exhaustion and turnover intentions	International Journal of Conflict Management		No		
2023	Shanmugapriya, J; Mehta, S; Saxena, T	Existential, relatedness and growth needs that determine turnover intention of rural CHC doctors in TAMIL NADU: Structural Equation Model	Journal of Health Management	No			
2023	Lee, Junghoon; Kim, Junekyu; Lim, Hong-A.; Song, Yeoungsuk	The mediating and moderating role of recovery experience between occupational stress and turnover intention in nurses caring for patients with COVID-19.	Journal of clinical nursing		No		
2023	Krsnik, Sabina; Erjavec, Karmen	Influence of sociodemographic, organizational, and social factors on turnover consideration among eldercare workers: a quantitative survey.	International Journal of Environmental Research and Public Health	No			
2023	Li, Tiantian; Guan, Lizheng; Zhang, Rui; Han, Youli; Guo, Rui; Li, Jun; Ma, Chengyu; Zhang, Ning; Fan, Yezhi; Huang, Yumei	Roles of doctor-patient relationship perception and job satisfaction in the impact of workplace violence on medical professionals' turnover intentions in the early phase of COVID-19: a cross-sectional study in China.	BMJ Open		No		
2024	Selesho, RA; Matjie, MA	Exploring turnover intentions of employees at a South African government education council	SA Journal of Human Resource Management	No			

Publication Year	Author	Title	Publication Title	Mediator and / or Moderator	Sample with active public servants in the executive branch?	VTI	Impossibility of the Article
2024	Emidy, MB	United states federal employee development in turbulent times: using job demands-resources theory to explain changes in perceived performance and turnover intention during the covid-19 pandemic	Review of Public Personnel Administration			No	
2024	Li, Xueying; Yang, Chenxin; Liu, Libing; Ding, Yuanlu; Xue, Jianchun; He, Jiani; Wu, Hui; Liu, Li	Configurational paths to turnover intention among primary public health workers in Liaoning Province, China: a fuzzy-set qualitative comparative analysis.	BMC public health	No			
2018	Yildiz, SM	An empirical analysis of the leader-member exchange and employee turnover intentions mediated by mobbing: evidence from sport organisations	Economic Research-Ekonomska Istrazivanja		No		
2019	Moon, KK; Park, J	Leadership styles and turnover behavior in the U.S federal government: does span of control matter?	International Public Management Journal			No	
2017	Sun, RS; Wang, WJ	Transformational leadership, employee turnover intention, and actual voluntary turnover in public organizations	Public Management Review			No	

Appendix C
ROBUST Analysis

Publication Year	Author	Title	Item 1	Item 2	Item 3	Item 4	Item 5	Item 6	Item 7	Yes criterion number
2008	Bright, L	Does public service motivation really make a difference on the job satisfaction and turnover intentions of public employees?	2	1	1	1	1	1	1	6
2008	Strolin-Goltzman, Jessica; McCarthy, Mary; Smith, Brenda; Caringi, Jim; Bronstein, Laura; Lawson, Hal	Should I stay or should I go? A comparison study of intention to leave among public child welfare systems with high and low turnover rates.	2	2	1	1	2	1	1	4
2009	Lai, L; Kapstad, JC	Perceived competence mobilization: an explorative study of predictors and impact on turnover intentions	2	2	2	1	1	1	2	3
2010	Liu, BC; Liu, JX; Hu, J	Person-organization fit, job satisfaction, and turnover intention: an empirical study in the Chinese public sector	2	2	1	1	1	1	1	5
2011	Pitts, D; Marvel, J; Fernandez, S	So hard to say goodbye? Turnover intention among U.S. federal employees	1	1	1	1	1	1	1	7
2013	Dysvik, A; Kuvaas, B	Perceived job autonomy and turnover intention: The moderating role of perceived supervisor support	2	1	2	1	1	1	1	5

Publication Year	Author	Title	Item 1	Item 2	Item 3	Item 4	Item 5	Item 6	Item 7	Yes criterion number
2014	Campbell, JW; Im, T; Jeong, J	Internal efficiency and turnover intention: evidence from local government in South Korea	1	1	1	1	1	1	1	7
2014	Ertürk, A	Influences of HR practices, social exchange, and trust on turnover intentions of public IT professionals	2	1	1	2	1	1	1	5
2015	Kim, J	What increases public employees’ turnover intention?	1	1	1	1	1	1	1	7
2015	Ertas, N	Turnover intentions and work motivations of millennial employees in federal service	1	1	1	1	1	1	1	7
2016	Caillier, JG	Do transformational leaders affect turnover intentions and extra-role behaviors through mission valence?	2	1	1	2	1	1	1	5
2016	Campbell, JW; Im, T	PSM and turnover intention in public organizations: does change-oriented organizational citizenship behavior play a role?	2	1	2	2	1	1	2	3
2016	Caillier, JG	Linking transformational leadership to self-efficacy, extra-role behaviors, and turnover intentions in public agencies: the mediating role of goal clarity	2	1	1	1	1	1	1	6
2017	Shim, DC; Park, HH; Eom, TH	Street-level bureaucrats' turnover intention: does public service motivation matter?	1	1	2	1	2	1	2	4
2017	Kim, SY; Fernandez, S	Employee empowerment and turnover intention in the U.S. federal bureaucracy	1	1	1	1	2	1	2	5
2017	Van Waeyenberg, T; Decramer, A;	The relationship between employee performance management and civil servants' turnover intentions: a test of the mediating roles of system satisfaction and affective commitment	2	2	2	1	1	1	2	3

Publication Year	Author	Title	Item 1	Item 2	Item 3	Item 4	Item 5	Item 6	Item 7	Yes criterion number
	Desmidt, S; Audenaert, M									
2018	Jabeen, F; Friesen, HL; Ghoudi, K	Quality of work life of Emirati women and its influence on job satisfaction and turnover intention: Evidence from the UAE	1	1	2	1	1	1	2	5
2018	Jin, MH; McDonald, B; Park, J	Person–organization fit and turnover intention: Exploring the mediating role of employee followership and job satisfaction through conservation of resources theory	2	2	2	1	1	1	1	4
2018	Hussain, T; Deery, S	Why do self-initiated expatriates quit their jobs: The role of job embeddedness and shocks in explaining turnover intentions	2	1	2	2	1	1	2	3
2018	Siyal, Saeed; Peng, Xiaobao	Does leadership lessen turnover? The moderated mediation effect of leader–member exchange and perspective taking on public servants.	2	2	2	2	1	1	2	2
2019	Sabharwal, M; Levine, H; D'Agostino, M; Nguyen, T	Inclusive work practices: turnover intentions among LGBT employees of the U.S. federal government	1	1	1	1	1	1	1	7
2019	Mustafa, G; Ali, N	Rewards, autonomous motivation and turnover intention: Results from a non-Western cultural context	2	2	2	2	1	1	2	2
2019	Kakar, AS; Abu Mansor, NN; Saufi, RA; Singh, H	Work-life balance practices and turnover intention: The mediating role of person-organization fit	2	2	2	1	1	1	1	4

Publication Year	Author	Title	Item 1	Item 2	Item 3	Item 4	Item 5	Item 6	Item 7	Yes criterion number
2019	Shareef, RA; Atan, T	The influence of ethical leadership on academic employees' organizational citizenship behavior and turnover intention Mediating role of intrinsic motivation	2	1	1	1	1	1	1	6
2021	Mullins, LB; Charbonneau, É; Riccucci, NM	The effects of family responsibilities discrimination on public employees’ satisfaction and turnover intentions: Can flexible work arrangements help?	1	1	1	1	2	1	1	6
2021	Samad, A; Memon, SB; Maitlo, AA	Workplace incivility and turnover intention among nurses of public healthcare system in Pakistan	2	1	2	1	1	1	1	5
2021	Bao, YJ; Zhong, W	Public service motivation matters: examining the differential effects of challenge and hindrance stressors on organizational identification and turnover intention	2	2	1	1	1	1	1	5
2021	Boon, J; Wynen, J; Kleizen, B	What happens when the going gets tough? Linking change scepticism, organizational identification, and turnover intentions	1	1	1	1	1	1	1	7
2021	Bright, L	Does perceptions of organizational prestige mediate the relationship between public service motivation, job satisfaction, and the turnover intentions of federal employees?	2	2	1	1	1	1	2	4
2021	Gadi, PD; Kee, DMH	Workplace bullying, human resource management practices, and turnover intention: the mediating effect of work engagement: evidence of Nigeria	2	2	1	1	1	1	2	4
2021	Ko, MC; Campbell, JW	Social capital, person-organization fit and cynicism: a multi-stage mediation model of turnover intention in public organizations	2	2	2	1	1	1	2	3

Publication Year	Author	Title	Item 1	Item 2	Item 3	Item 4	Item 5	Item 6	Item 7	Yes criterion number
2021	Ma, SZ; Xu, XH; Trigo, V; Ramalho, NJC	Managing doctor-patient relationships and turnover intention in Chinese hospitals with commitment HRM: The moderating role of pragmatism	2	2	2	1	1	1	2	3
2021	Barkhuizen, NE; Gumede, B	The relationship between talent management, job satisfaction and voluntary turnover intentions of employees in a selected government institution	2	1	2	1	1	1	1	5
2021	Bas, M; Çinar, O	The mediating role of work engagement in the relationship between perceived organizational support and turnover intention – with an application to healthcare employees in Erzincan province of Turkey	1	1	1	1	1	1	2	6
2022	Chordiya, R	A study of interracial differences in turnover intentions: the mitigating role of pro-diversity and justice-oriented management	1	1	1	1	1	1	1	7
2022	Li, Y; Xie, W	Linking change-oriented organizational citizenship behavior to turnover intention: Effects of servant leadership and career commitment	2	2	1	1	1	1	2	4
2022	Chordiya, R	Organizational inclusion and turnover intentions of federal employees with disabilities	1	1	1	1	1	1	1	7
2022	Lin, CY; Huang, CK; Li, HX; Chang, TW; Hsu, YC	Will they stay or leave? Interplay of organizational learning culture and workplace mindfulness on job satisfaction and turnover intentions	2	2	1	1	1	1	2	4
2022	Albtoosh, Q; Ngah, AH; Yusoff, YM	Training satisfaction relative to turnover intention: the mediating role of employee loyalty	2	2	1	1	1	1	1	5

Publication Year	Author	Title	Item 1	Item 2	Item 3	Item 4	Item 5	Item 6	Item 7	Yes criterion number
2022	Akhtar, Muhammad Sajid; Tatlah, Ijaz Ahmad	Effect of organizational justice on turnover intention through job satisfaction among teacher educators in public universities	2	2	2	1	2	1	2	2
2022	Delgado-Bello, Cristian A.; Gahona Flores, Orlando F.	Impacto del liderazgo transformacional en la satisfacción laboral y la intención de abandono: un estudio desde el contexto educativo.	2	2	2	1	1	1	2	3
2023	Bao, YJ; Zhong, W	Public service motivation helps: Understanding the influence of public employees' perceived overqualification on turnover intentions	2	2	1	1	1	1	1	5
2023	Mussagulova, A; Demircioglu, MA	What affects the turnover intention of civil servants: Evidence from Bhutan	2	2	2	1	1	2	2	2
2023	Nguyen, TD; Nguyen, TT; Nguyen, PC	Job embeddedness and turnover intention in the public sector: the role of life satisfaction and ethical leadership	2	2	1	1	1	1	2	4
2023	Wirawan, H; Salam, R; Normawati, N; Paramarta, V; Sunarsi, D	Predicting turnover intention in Indonesian public organisations: investigating the effect of citizen and workplace incivility, and job insecurity	2	2	1	1	1	1	1	5
2023	Wesemann, A	Turbulence Ahead: Strategic Human Capital Management, Job Satisfaction, and Turnover Intention	1	1	1	1	1	1	2	6

Publication Year	Author	Title	Item 1	Item 2	Item 3	Item 4	Item 5	Item 6	Item 7	Yes criterion number
2023	Nguyen, NTH; Nguyen, D; Teo, S; Xerri, MJ	Abusive supervision and turnover intention among public servants: the roles of psychological distress and person-organization fit	2	2	2	1	1	1	1	4
2023	Al-Mahdy, YFH; Alazmi, AA	Principal support and teacher turnover intention in Kuwait: implications for policymakers	2	1	2	1	1	1	2	4
2023	Diko, TK; Saxena, S	Mediating role of employee engagement with transformational leadership and turnover intention	2	1	2	1	1	1	1	5
2023	Supi; Noermijati; Irawanto, DW; Puspaningrum, A	Talent management practices and turnover intention: The role of perceived distributive justice and perceived organizational support	2	1	1	1	1	1	2	5
2023	Senapaty, S; Venugopal, P	When do personal factors make autonomy motivational orientation worthwhile? A case of turnover intentions	2	2	1	1	1	1	1	5
2023	Dahle, DY; Urstad, SS	Judge and fury: instrumentality, feedback formats and turnover intention in schools	2	2	2	1	1	1	2	3
2023	Aman-Ullah, A; Ali, A; Ariza-Montes, A; Mehmood, W; Saraih, UN	Nexus of workplace incivility, workplace violence and turnover intentions: a mediation study through job burnout	2	2	1	1	1	1	1	5
2023	Zhang, HY; Shi, YY; Teng, LS	Exploring relationships of job satisfaction and burnout with turnover intention among Chinese English language teachers	1	1	1	1	1	1	1	7
2023	Xu, Z; Zhang, L; Yang, Z; Yang, G	Burnout and turnover intention of primary health care providers during the COVID-19 pandemic in China	2	2	1	1	1	1	1	5

Publication Year	Author	Title	Item 1	Item 2	Item 3	Item 4	Item 5	Item 6	Item 7	Yes criterion number
2023	Kakar, AS; Misron, A; Rauza; Meyer, N; Durrani, DK	Job insecurity as a mediator between fearing COVID-19 and turnover intention: empirical evidence during the COVID-19 pandemic	2	2	1	1	1	1	1	5
2023	Liu, Y; Yu, YP; Zeng, XY; Li, YF	Linking preschool teachers' pay equity and turnover intention in Chinese public kindergartens: The mediating role of perceived organizational support and job satisfaction	2	2	1	1	1	1	1	5
2023	Yucel, I; Sirin, MS; Bas, M	The mediating effect of work engagement on the relationship between work-family conflict and turnover intention and moderated mediating role of supervisor support during global pandemic	1	1	2	1	2	1	1	5
2023	Obuobisa-Darko, Theresa; Sokro, Evans	Psychological impact of COVID-19 pandemic and turnover intention: The moderating effect of employee work engagement.	2	2	1	2	1	1	1	4
2023	Lim, Jaeyoung; Moon, Kuk-Kyoung	Exploring the effect of emotional labor on turnover intention and the moderating role of perceived organizational support: Evidence from Korean firefighters	2	2	2	1	2	1	2	2

Legend: Item 1. Is the sampling frame largely representative? Item 2. Were appropriate participant recruitment methods utilized? Item 3. Is the exclusion rate acceptable? Item 4. Is the final sample size sufficient? Item 5. Are demographic variables reported? Item 6.Do the measures have adequate reliability? Item 7.Was management of data acceptable? 1 = yes; 2 = no
